# Combined Effects of Ventilation Mode and Positive End-Expiratory Pressure on Mechanics, Gas Exchange and the Epithelium in Mice with Acute Lung Injury

**DOI:** 10.1371/journal.pone.0053934

**Published:** 2013-01-09

**Authors:** Apiradee Thammanomai, Hiroshi Hamakawa, Erzsébet Bartolák-Suki, Béla Suki

**Affiliations:** Department of Biomedical Engineering, Boston University, Boston, Massachusetts, United States of America; University of Adelaide, Australia

## Abstract

The accepted protocol to ventilate patients with acute lung injury is to use low tidal volume (V_T_) in combination with recruitment maneuvers or positive end-expiratory pressure (PEEP). However, an important aspect of mechanical ventilation has not been considered: the combined effects of PEEP and ventilation modes on the integrity of the epithelium. Additionally, it is implicitly assumed that the best PEEP-V_T_ combination also protects the epithelium. We aimed to investigate the effects of ventilation mode and PEEP on respiratory mechanics, peak airway pressures and gas exchange as well as on lung surfactant and epithelial cell integrity in mice with acute lung injury. HCl-injured mice were ventilated at PEEPs of 3 and 6 cmH_2_O with conventional ventilation (CV), CV with intermittent large breaths (CV_LB_) to promote recruitment, and a new mode, variable ventilation, optimized for mice (VV_N_). Mechanics and gas exchange were measured during ventilation and surfactant protein (SP)-B, proSP-B and E-cadherin levels were determined from lavage and lung homogenate. PEEP had a significant effect on mechanics, gas exchange and the epithelium. The higher PEEP reduced lung collapse and improved mechanics and gas exchange but it also down regulated surfactant release and production and increased epithelial cell injury. While CV_LB_ was better than CV, VV_N_ outperformed CV_LB_ in recruitment, reduced epithelial injury and, via a dynamic mechanotransduction, it also triggered increased release and production of surfactant. For long-term outcome, selection of optimal PEEP and ventilation mode may be based on balancing lung physiology with epithelial injury.

## Introduction

In acute respiratory distress syndrome (ARDS), the currently accepted protocol is to use low tidal volume (V_T_) in combination with a procedure that helps keep the lung open such as a recruitment maneuver (RM) or adding positive end-expiratory pressure (PEEP) [1]. PEEP stabilizes the injured alveoli and prolongs the effects of RM such as sustained high airway pressure [2]. However, PEEP can also decrease cardiac output [3], or increase pulmonary edema [4]. Furthermore, ventilation superimposed on a high PEEP can lead to ventilator induced lung injury (VILI) due to barotrauma and/or volutrauma [5].

The development of VILI is related to the heterogeneous nature of ARDS lungs [6]. When a high PEEP is required to maintain an open lung, normal lung regions will be overinflated. In contrast, an inadequate PEEP can result in cyclic recruitment/derecruitment during ventilation with high non-physiologic shear and normal stresses on the epithelium which can generate epithelial injury [7], [8]. Consequently, the selection of an optimal PEEP is a highly debated topic [9]–[13]. A moderate PEEP together with regular delivery of large breaths as RMs might be able to keep the lung open while minimizing the risk of VILI. This approach works in mice [14]–[16] but clinical studies showed mixed results [10], [17], [18].

Variable ventilation (VV) introduced by Lefevre et al. [19] better maintains an open lung than conventional ventilation (CV) [20]–[27]. Recently, we introduced a new optimized VV (VV_N_) for both normal and HCl-injured mice which significantly improved respiratory mechanics and oxygenation over several other methods without causing additional injury [27]. Since both PEEP and RM have a significant impact on ventilator performance, the aim of the current study was to investigate the combined effects of ventilation mode and PEEP on physiology including mechanics and gas exchange and on lung biology including surfactant and epithelial cell integrity in mice with acute lung injury (ALI) at two PEEP levels. To this end, HCl-injured mice were ventilated with CV, CV with intermittent large breaths (CV_LB_) to promote recruitment, and VV_N_ at PEEPs of 3 and 6 cmH_2_O. While the raw physiological data including mechanics and gas exchange at 3 cmH_2_O PEEP were reported in our previous paper [27], the mechanics data were reanalyzed using an advanced model. Additionally, all the biochemical data reported here are novel.

## Methods

### Animal Preparation

The protocol was approved by the Animal Care and Use Committee of Boston University (approval number: 04–033). Male C57BL/6 mice (weight: 22–26 g, Charles River Laboratories, Wilmington, MA) were used throughout the studies. The mice were anesthetized with an intraperitoneal injection of 70 mg/kg of pentobarbital sodium, tracheostomized with an 18-guage metal cannula and placed on a heated pad to maintain a constant body temperature (37°C) throughout the experiment. Extra doses of pentobarbital sodium (20 mg/kg) were administered every 20 minutes to keep the animal in a deeply anesthetized state and minimize any suffering. The tracheal cannula was later connected to the outlet of a small animal ventilator-oscillator system (flexiVent ventilator, SCIREQ, CA).

### Acid Aspiration Lung Injury

In order to obtain a mouse model of lung injury, hydrochloric acid (HCl, 0.1 M, pH = 1.25) was introduced intratracheally in 1 µL/g increments with a bolus of air in between for a total of 3 µL/g. To prevent lung collapse, the mice were connected to the ventilator, immediately given 2 RMs defined as a ramp increase in volume to 1 ml in 4 sec, and subsequently 1 RM after 5 and 10 minutes. The animals were then ventilated using a constant V_T_ of 8 ml/kg for an additional period of 20 minutes at a PEEP of 3 cmH_2_O. The dose and the delivery method were developed and tested in a pilot study using 6 mice.

### Ventilation Protocols

We aimed to investigate the physiological effects of each ventilation mode at 2 levels of PEEP using 3 ventilation modes that were identical to those in our previous study in which actual time series of V_T_ are also shown [27]:

#### CV group

Mice were ventilated with a constant V_T_ of 8 ml/kg and a breathing frequency (f) of 240 breaths per minute.

#### VV_N_ group

Mice were ventilated with a ventilation mode that was designed previously [27]. Briefly, the shape of the V_T_ distribution is flat for small V_T_s followed by a power law decrease for larger V_T_s. The mean V_T_ (V_MEAN_) was set to 8 ml/kg and f was also adjusted to obtain constant minute ventilation on a cycle-by-cycle basis. The parameters specifying the distribution of V_T_ were as follows: the smallest V_T_ was V_MIN_ = 0.7 V_MEAN_; the V_T_ at which the distribution changes to a power law (with an exponent α = 5.1) was V_P_ = 0.9V_MEAN_. The maximum delivered V_T_ (V_MAX_) was set to 2.25 times the V_MEAN_. The median V_T_ for VV_N_ was 7.3 ml/kg.

#### CV_LB_ group

Mice were ventilated with a V_T_ of 8 ml/kg using a constant frequency at 240 breaths per minute. Twice in every minute, the animals received a large breath. Similar to VV_N_, f was reduced for each large breath to maintain constant minute ventilation. The size of the two large breaths was matched with the 2 largest V_T_ values of the VV_N_ mode in 1 minute.

**Figure 1:**
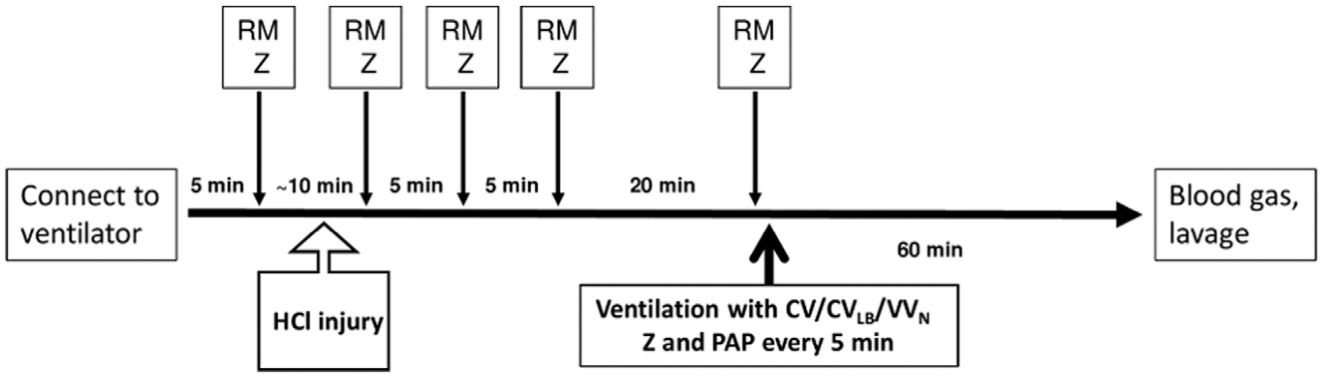
Time course of the protocol. Once the animal was connected to the ventilator, a recruitment maneuver (RM) was followed by impedance measurement (Z) and HCl treatment. After a stabilization period and several RM and Z, a 60 min ventilation period (thick arrow) was started. During the ventilation period, Z and peak airway pressure (PAP) were recorded at 5 min intervals. At the end of the protocol, blood gases and a lavage sample were obtained and the lung was isolated for further processing.

The time course of treatment and ventilation is illustrated in Fig. 1. A total of 48 animals were used with 8 mice in each ventilation group, 3 ventilation and 2 PEEP groups. At the beginning of the ventilation protocol, animals received 2 RMs to standardize volume history and then ventilated with room air for 60 minutes with CV mode, VV_N_ mode or CV_LB_ mode. The desired PEEP, 3 or 6 cmH_2_O, was maintained by placing the expiratory port of the ventilator in a water trap. For the animals to be ventilated at a PEEP of 6 cmH_2_O, the PEEP level was adjusted immediately after 2 RMs at the beginning of the protocol and the baseline mechanics were measured.

#### Unventilated group

Additionally, 4 animals received HCl injury and the initial treatment as shown in Fig. 1. However, these animals were sacrificed just before the 60 min ventilation protocol started. The lungs of these animals were lavaged and homogenized for biochemical analysis (see below) and represent the state of the lung at the beginning of the 60 min ventilation protocol.

### Impedance Measurement

Respiratory mechanics were determined by the force oscillation technique by using the Optimum Ventilation Waveform [28]. Airway opening pressure and flow were obtained from the flexiVent ventilator and the data were processed offline using Fourier analysis to obtain respiratory input impedance (*Z_RS_*) every 5 minutes throughout the ventilation protocol.

### Model Description and Parameter Estimation

Respiratory input impedance *Z_RS_* was fitted by the single compartment constant-phase model [29] and a heterogeneous model [30], which characterizes heterogeneity of tissue elasticity of the lung that invariably occurs after lung injury [31]. Briefly, the constant-phase model characterizes tissue impedance by a tissue damping coefficient (*G*) and a tissue elastance coefficient (*H*) while the airway structure is partitioned to airway resistance (*R_aw_*) and inertance (*I_aw_*). For the total respiratory system, the chest wall also contributes to the resistance and the model provides an estimate of the total Newtonian resistance (*R*). The heterogeneous tissue model represents the airway tree by a set of parallel pathways, each composed of *R_aw_* and *I_aw_* and a tissue compartment. The values of *R_aw_* and *I_aw_* are assumed to be the same for every pathway whereas each tissue compartment is described by the constant-phase model. The *H* is assumed to be distributed according to a probability density function *n(H)* which is proportional to *1/H* between a minimum (*H_min_*) and a maximum (*H_max_*) value of H. In this study, only the values of *R*, *H*, *H_min_*, *H_max_* and the standard deviation (SD) of *n(H)* are reported. All parameters were estimated using a global optimization algorithm which minimized the root-mean squared (RMS) error between data and model [32].

### Airway Pressure Measurement

The airway pressure was monitored and recorded throughout the experiments using a separate pressure transducer (World Precision Instruments, Sarasota, FL) attached to the tracheal cannula. The mean peak airway pressure was calculated for every 5 minute period.

### Sample Processing

At the conclusion of the 60 min ventilation, arterial blood was collected from the carotid artery by clamping both the upper and lower parts with small clamps. A small incision was made between the clamps. The lower clamp was then released and the arterial blood was collected using capillary tubes and immediately analyzed for partial pressure of oxygen (PaO_2_), partial pressure of carbon dioxide (PaCO_2_), pH, and percent oxygen saturation (%sO_2_) using an I-STAT® blood gas analyzer (Abbott Laboratories, Abbott Park, Illinois, USA).

Lavage samples were also collected at the conclusion of the ventilation by instilling 1 ml of warm saline (37°C) via the tracheal cannula and slowly retrieving approximately 0.9 ml. The lavage sample was centrifuged and the cell-free supernatant was frozen until further analysis. The lung was then removed and homogenized in 2 ml of PBS at a pH of 7.2 and centrifuged. Supernatant was transferred to microcentrifuge tubes and stored frozen until further analysis.

### Total Protein and Western Blot Analysis

The amount of protein in the lavage and homogenate samples was measured using BCA protein assay reagent kit (Pierce, Rockford, IL). Equal amounts of total protein (7.8 µg) or equal volume (15 µl) of samples were separated using 4–20% SDS-polyacrylamide gels and transferred onto polyvinylidene fluoride membranes (Millipore, Bedford, MA). To test whether the ventilation mode had an effect on epithelial injury, Western blot analysis was carried out for E-cadherin from the lavage samples using a primary antibody for E-cadherin (CHEMICON, Temecula, CA). To test whether the ventilation mode had an effect on surfactant release and production, Western blot analysis was carried out for surfactant protein B (SP-B) from lavage and its proprotein form (proSP-B) (CHEMICON, Temecula, CA) from the homogenate samples. Densitometry was performed after chemiluminescence detection.

### Statistical Analysis

The statistical differences among parameters at different conditions were tested using t-tests and analyses of variance. For example, we used one-way ANOVA to compare parameters at 60 min, one-way repeated measure ANOVA to analyze time courses, two-way ANOVA to test the effects of PEEP and ventilation mode and their interactions. For post-hoc analyses, we used Tukey pairwise comparison. When data were not normally distributed, one way-ANOVA on ranks was used to analyze statistical differences. A value of p<0.05 was used to establish statistical significance. (SigmaStat, SPSS Inc, Chicago, IL).

## Results

**Figure 2:**
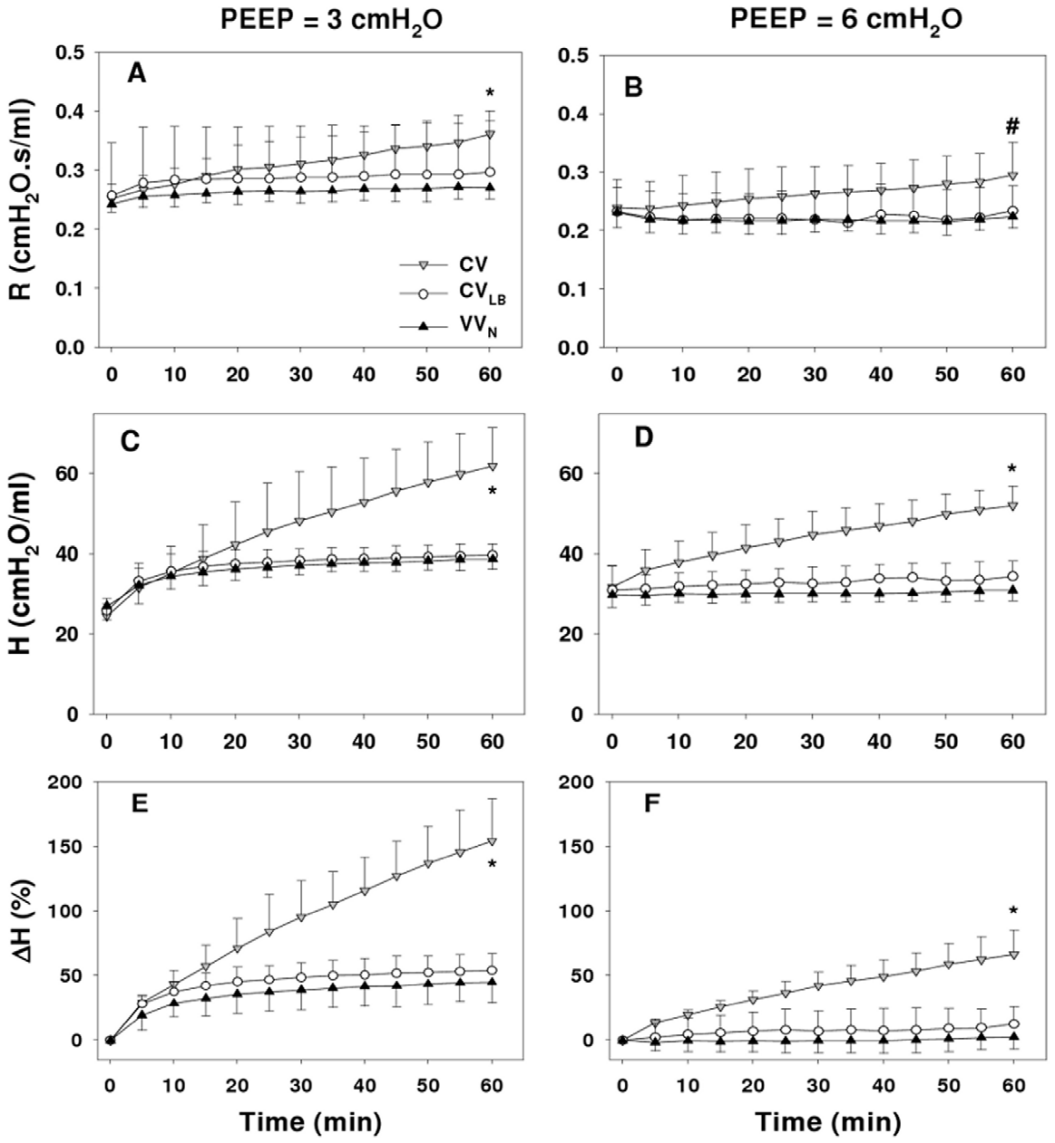
Mechanical parameters from the single compartment model. The graphs compare the time courses of Newtonian resistance (*R,* panels A and B), tissue elastance (*H,* panels C and D) and the change in H (*ΔH*, panels E and F) during 60 min of ventilation using conventional ventilation (CV), conventional ventilation with large breaths (CV_LB_), or variable ventilation (VV_N_) in HCl-injured mice at PEEPs of 3 (left panels) and 6 cmH_2_O (right panels). * denotes significant difference between CV and CV_LB_ as well as CV and VV_N_ at 60 min; # denote significant difference between CV and VV_N_ at 60 min. Additional significance levels are given in the text.

The time course of mechanical parameters in the HCl-injured mice ventilated at PEEPs of 3 cmH_2_O (PEEP3) and 6 cmH_2_O (PEEP6) are shown in Fig. 2. All parameters, *R* and *H* and the percent change in *H* (*ΔH*), depended on time (p<0.001). The *ΔH* during CV increased linearly while it reached a plateau during VV_N_ and CV_LB_. At 60 min, all parameters, *R, H* and *ΔH,* depended on both the ventilation mode (p<0.001) and PEEP (p<0.001). At PEEP3, R in the CV group was significantly higher than in the VV_N_ and CV_LB_ groups (p = 0.002 and p = 0.028, respectively) whereas at PEEP6, R during CV was significantly higher than during VV_N_ (p = 0.012), but not during CV_LB_. Both *H* and *ΔH* during CV was higher than during VV_N_ and CV_LB_ (p<0.001) at both PEEPs while there was no difference between *ΔH* during VV_N_ and CV_LB_.

**Figure 3:**
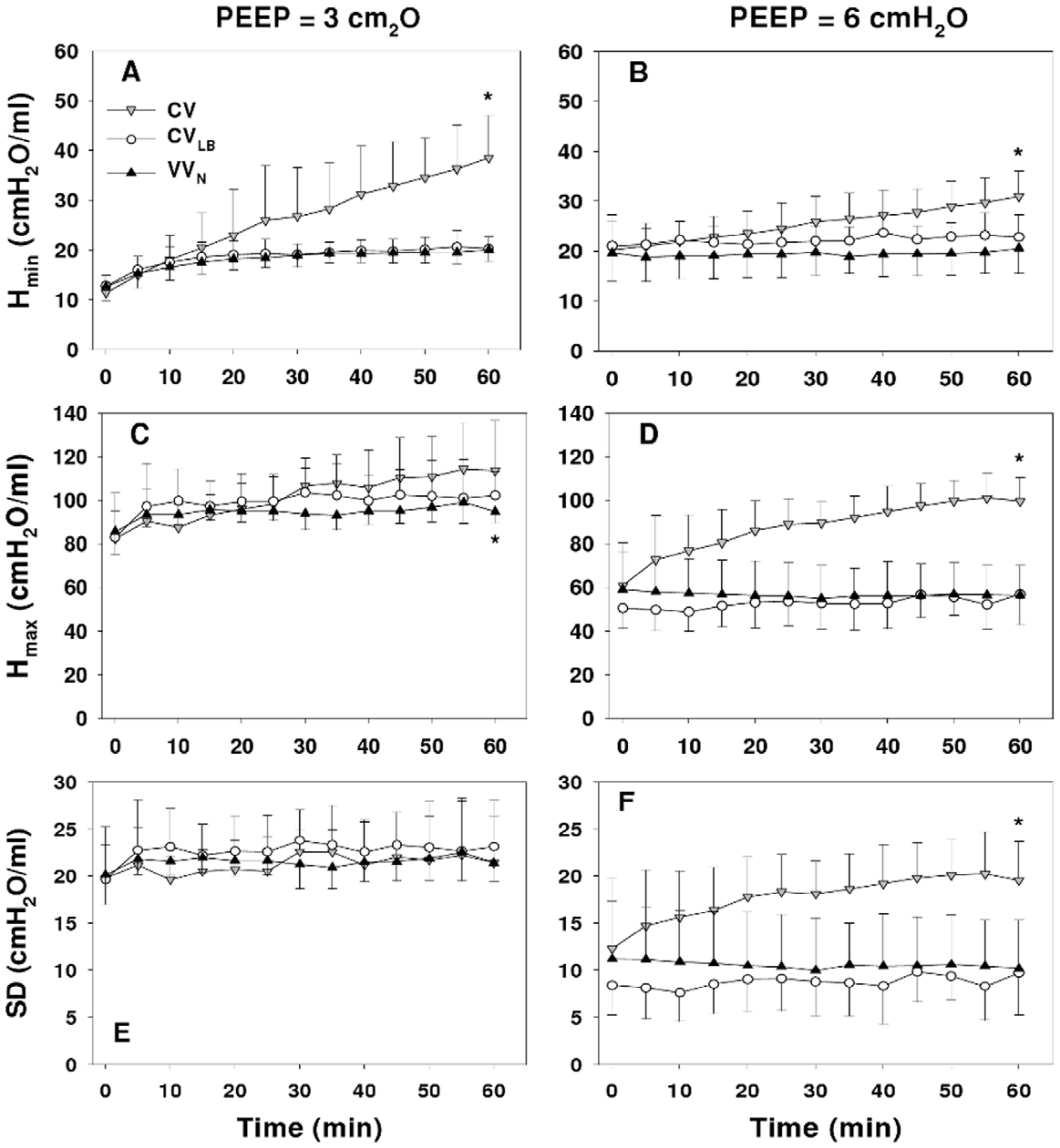
Mechanical parameters from the distributed model. The graphs compare the time courses of minimum (*H_min_*, panels A and B), maximum (*H_max_*, panels C and D) and standard deviation (SD, panels E and F) of the distribution of elastance in the heterogeneous tissue model during 60 min of ventilation using CV, CV_LB_ or VV_N_ in HCl-injured mice at PEEPs of 3 (left panels) and 6 cmH_2_O (right panels). * denotes significant difference (p<0.001) between CV and CV_LB_ as well as CV and VV_N_ at 60 min.

The *H_min_* (Fig. 3) describing the lowest regional stiffness increased with all ventilation modes at both PEEP3 (p<0.001) and PEEP6 (p<0.001 for CV, and p<0.05 for CV_LB_ and VV_N_). The *H_max_* describing the stiffest region increased only during CV and CV_LB_ (p<0.001) at PEEP3 whereas at PEEP6, it increased only during CV (p<0.001). The SD of *H* representing heterogeneity of regional lung stiffness increased only during CV at PEEP6 (p<0.001). At 60 min, both *H*_min_ and *H*_max_ were higher during CV than during CV_LB_ and VV_N_ (p<0.001). At PEEP6, the SD of *H* during CV at 60 min was also different from that during CV_LB_ and VV_N_ (p<0.001).

**Figure 4:**
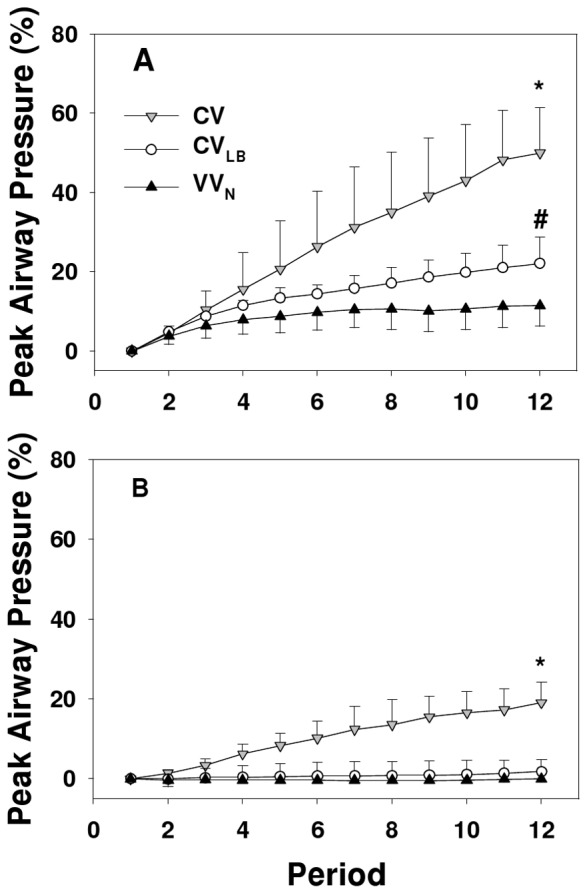
Peak airway pressures as a function of ventilation mode. The graphs compare the time courses of the relative percentage change in mean peak airway pressure during 60 min of ventilation using CV, CV_LB_ or VV_N_ in HCl-injured mice at PEEPs of 3 (panel A) and 6 cmH_2_O (panel B). Each point is calculated from the average of the peak airway pressure in a 5–minute ventilation period compared to its value at time 0. *denotes significant difference (p<0.001) between CV and CV_LB_ as well as CV and VV_N_ at 60 min; # denote significant difference (p<0.02) between CV_LB_ and VV_N_ at 60 min.

At time 0, the mean peak airway pressure (*_PAP_*) started at 10 and 14 cmH_2_O at PEEP3 and PEEP6, respectively. By 60 min during CV, *PAP* reached 14 and 18 cmH_2_O at PEEP3 and PEEP6, respectively. The corresponding percent increases in PAP (*ΔPAP*) during CV_LB_ and VV_N_ were much smaller than during CV (Fig. 4). For CV and CV_LB_ at both PEEPs, *ΔPAP* was significantly affected by time (p<0.001) whereas during VV_N_, *ΔPAP* depended on time only at PEEP3 (p<0.001). The *PAP* at 60 min was significantly lower during VV_N_ and CV_LB_ than during CV (p<0.005). At 60 min, *ΔPAP* was significantly affected by both ventilation mode and PEEP (p<0.001) and it was higher during CV than VV_N_ and CV_LB_ (p<0.001). At 60 minutes, *ΔPAP* at PEEP3 was higher during CV_LB_ than during VV_N_ (p<0.02).

**Figure 5:**
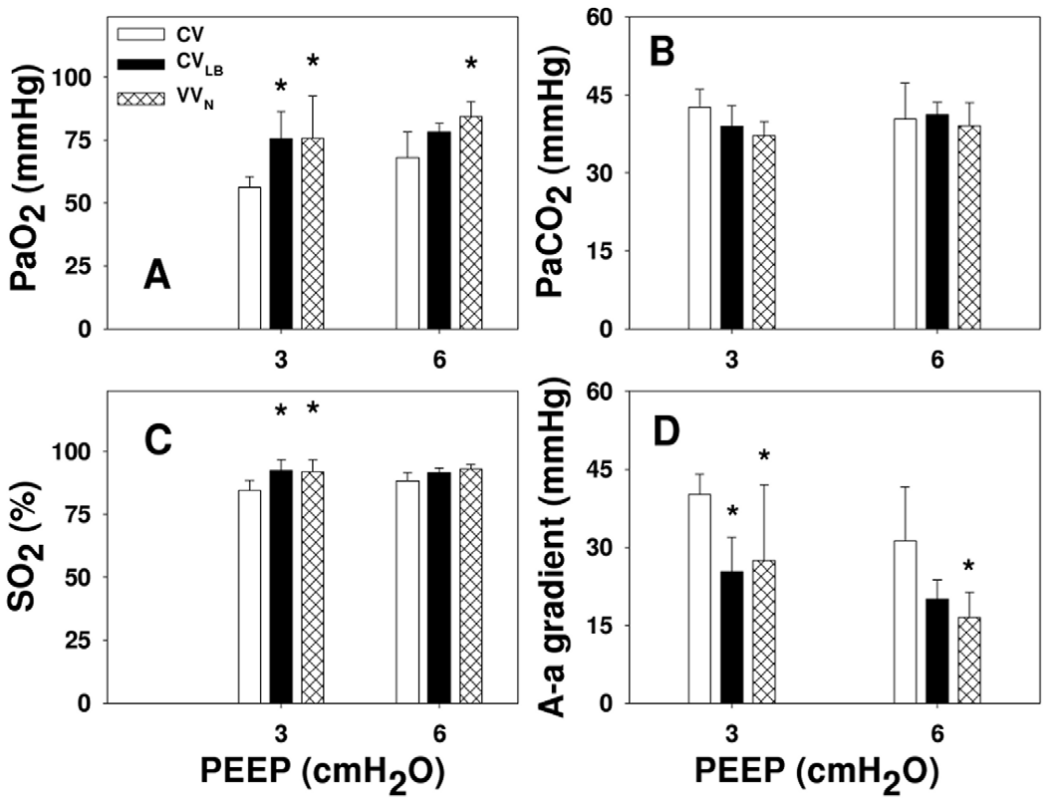
Gas exchange as a function of ventilation mode. The graphs show the partial pressures of oxygen and carbon dioxide (PaO_2_ and PaCO_2_, respectively, in panels A and B), percent oxygen saturation (SO_2_, panel C) and Alveolar-arterial gradient (A–a gradient, panel D) obtained at the end of 60 min ventilation using CV, CV_LB_ or VV_N_ in HCl-injured mice at PEEPs of 3 and 6 cmH_2_O. * denotes significant difference compared to CV (p<0.05).

The PaO_2_, %sO_2_ as well as A-a gradient were affected by both ventilation (p<0.001) and PEEP (p<0.03) (Fig. 5). At PEEP3, PaO_2_ and %sO_2_ were higher during VV_N_ and CV_LB_ compared to CV (p<0.005 and p<0.02, respectively). At PEEP6, PaO_2_ during VV_N_ was higher than during CV (p = 0.012). Furthermore, at PEEP3, A-a gradient during CV was higher than during VV_N_ and CV_LB_ (p<0.05), while at PEEP6, the A-a gradient was higher during CV than VV_N_ (p = 0.019).

**Figure 6:**
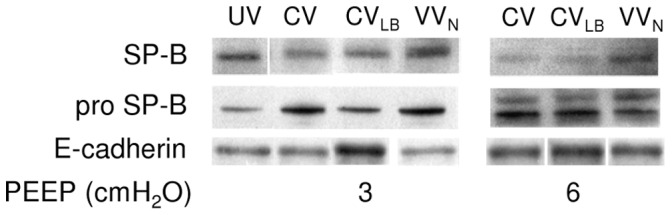
Example Western blots. Representative blots are shown for surfactant protein (SP)-B and its pro form (proSP-B) as well as E-cadherin obtained from lung homogenates and lavage fluid at the conclusion of 60 min of ventilation using CV, CV_LB_ or VV_N_ at PEEPs of 3 (left) and 6 cmH_2_O (right). UV denotes blots from HCl-injured but unventilated group of animals. Note the small gaps between several images for a given protein. These blots are from the same film, but not in the same order as the rest of the blots. The original image was first cut into pieces, without changing the image, and then reassembled in the desired order.

**Figure 7:**
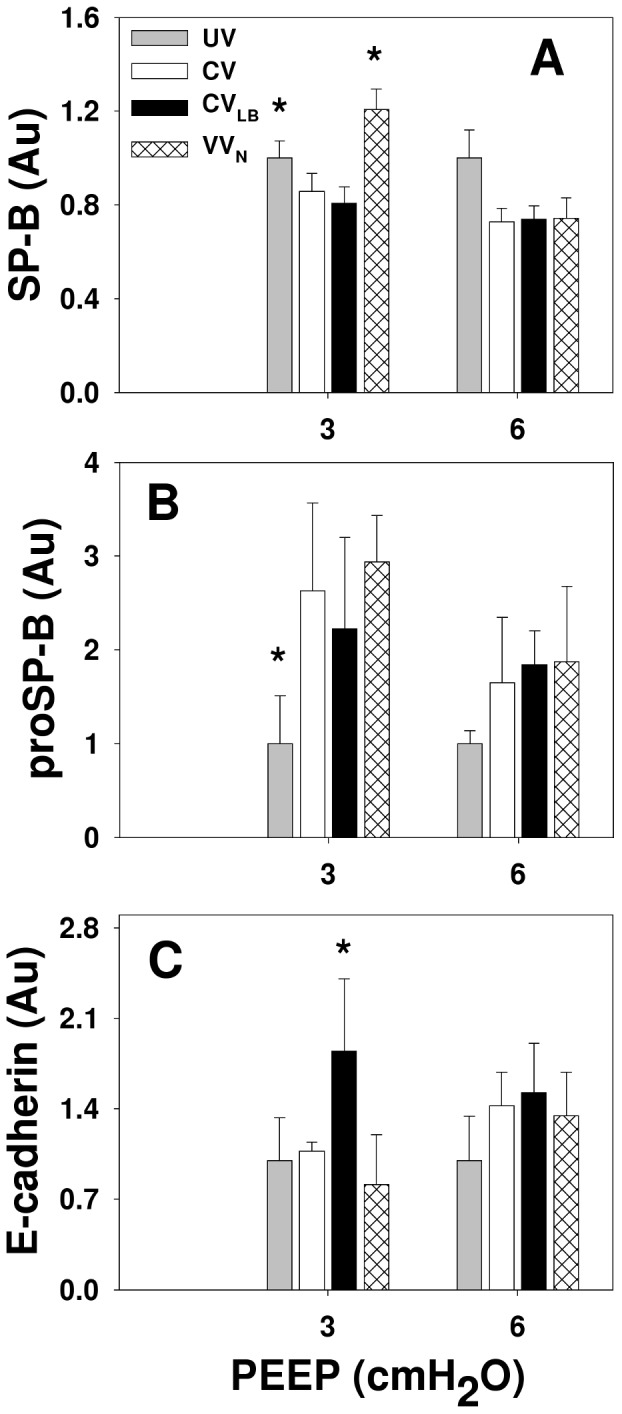
Analysis of Western blots. Graphs show the relative amounts of SP-B, proSP-B and soluble E-cadherin in HCl-injured mice at the conclusion of 60 min of ventilation using CV, CV_LB_ or VV_N_ at PEEPs of 3 and 6 cmH_2_O. The data are normalized with the corresponding mean values of the unventilated group with baseline injury. The two bars in the unventilated group at the two PEEP levels correspond to samples from the same lungs on two separate Western blots. * denotes significant difference compared to CV (p<0.05).

Example Western blots for SP-B, proSP-B and E-cadherin are demonstrated in Fig. 6 and group means normalized to the mean of the unventilated groups are given in Fig. 7. At PEEP3, SP-B was significantly higher than at PEEP6 (p<0.001) and while CV and CV_LB_ had lower levels than the unventilated group (p<0.001), VV_N_ had a higher level than any other group (p<0.001). The proSP-B also significantly depended on PEEP (p = 0.004). At PEEP3, animals during CV and VV_N_, but not CV_LB_, had higher proSP-B than in the unventilated group (p = 0.006 and p = 0.001, respectively). For E-cadherin, we detected its 85 kDa soluble form in the lavage with significant interaction between PEEP and ventilation (p<0.03). During CV and VV_N_, the E-cadherin was higher at PEEP6 than at PEEP3 (p<0.05) whereas at PEEP3, E-cadherin during CV_LB_ was higher than during CV, VV_N_ (p<0.05) and the unventilated (p<0.003) groups. Excluding the unventilated groups, there was no difference in any of these expressions among the ventilation groups at PEEP6.

## Discussion

The lower inflection point (LIP) on the pressure-volume (P-V) curve is often thought to represent alveolar recruitment and setting the PEEP just above LIP can improve lung function in patients with ARDS and increase their chances of early weaning [33]. However, Mergoni et al. showed that LIP is not a good indicator of alveolar recruitment [34]. Indeed, while the regional P-V curves can exhibit behavior consistent with recruitment, the total P-V curve may not show an LIP [9]. It is generally accepted that high V_T_ and plateau airway pressure lead to lung injury [35] via exposing the epithelium to large stresses and strains [36]. Recently, Chiumello et al. [37] showed that V_T_ and airway pressure are not adequate surrogates of lung stress and strain due to the large heterogeneity of lung stiffness in ARDS patients in agreement with the above notion that regional heterogeneity is not reflected in the total P-V curve. A mathematical index of stress and strain was introduced by Brunner and Wysocki [38] to predict the best combination of V_T_ and f as a function of PEEP. Other methods include titrating the PEEP for best gas exchange [39] or using dynamic features derived from respiratory mechanics [9], [11], [31].The general findings are that within some limits, a higher PEEP is better in terms of lung function as it maintains the lung open.

The above studies do not address two aspects of mechanical ventilation. The first is the combined effects of PEEP and less conventional modes of ventilation such as CV with RM and VV on physiology. The second is that these studies do not consider epithelial mechanobiology. It is implicitly assumed that the PEEP-V_T_ combination best for physiological outcome also protects the epithelium. In this study, we compared the effects of three ventilation modes on physiology in HCL-injured mice at two PEEP levels. We also investigated the effects of ventilation mode on SP-B that is important in maintaining low surface tension as well as epithelial cell injury characterized by E-cadherin. The main results are that *1*) ventilation performance was PEEP dependent; *2)* the performance of VV_N_ and CV_LB_ was significantly better than that of CV, the current clinical method; *3)* there were small, but consistent improvements in lung physiology during VV_N_ compared to CV_LB_; 4) despite better physiology including mechanical parameters, airway pressures and gas exchange at the higher PEEP, the lower PEEP was advantageous in terms of surfactant and epithelial cell integrity; and 5) VV_N_ was superior to CV_LB_ in terms of epithelial mechanobiology.

### Effects of PEEP on Ventilation Performance

The effects of PEEP on lung physiology and biology have been studied in numerous animal models of lung injury [8], [11], [15], [16], [40]–[43]. In our study, we compared the performance of three ventilation modes at PEEP3 and PEEP6 that are well tolerated by the mouse during injury [14], [15]. The PEEP3 was a necessary minimum PEEP because in a preliminary study, we found that mice with HCl injury did not tolerate the 60 min ventilation at a PEEP of 1 cmH_2_O. The PEEP6 in mice may correspond to a PEEP of about 15 cmH_2_O in humans. The reason is as follows. The *in situ* transpulmonary pressure of the mouse lung is between 1.5 and 2 cmH_2_O and PEEP6 is about 3–4 times higher than this range. In humans, the average transpulmonary pressure is around 5 cmH_2_O. Thus, our PEEP6 in the mouse might correspond to a PEEP of 15–20 cmH_2_O PEEP in human patients, not uncommon in the ICU. However, the peak airway pressures at PEEP6 barely reached 20 cmH_2_O even during CV. Allen et al. [14] investigated the effects of deep inspiration on lung mechanics and found a strong PEEP dependence albeit during short term ventilation. Since the effects of PEEP were studied in the same animals, it is possible that the results at different PEEP levels were not independent of each other. This was ruled out here because each mouse was ventilated for an hour at a single PEEP.

#### Respiratory mechanics

Using the constant phase model (Fig. 2), all parameters were highly PEEP dependent consistent with the data of Allen et al. in that the recovery in *H* over a 7-min period after a large breath was inversely related to PEEP between 1 and 6 cmH_2_O in both acid and endotoxin models of ALI [14]. All parameters during CV substantially increased with time suggesting massive derecruitment. In contrast, respiratory mechanics at PEEP3 during VV_N_ or CV_LB_ reached their respective plateau levels after 30 min implying that CV_LB_ and VV_N_ maintained the lung open albeit at different levels.

In the heterogeneous model (Fig. 3), only *H*_max_ and SD were PEEP dependent. Although *H_min_* was not PEEP dependent, its percent increase highly depended on PEEP with a rate much slower at the higher PEEP. This is perhaps not surprising, since a higher PEEP prevents or slows down the collapsing process [2]. At PEEP6, there was no change in *ΔPAP* during VV_N_. Thus, PEEP significantly affected the rate of lung collapse in a ventilation mode dependent manner. Further, in agreement with Chiumello et al. [37], the SD of *H* increased over time during CV becoming significantly larger than during CV_LB_ or VV_N_. Such increase in regional lung stiffness is directly related to the heterogeneity of regional strain and hence is at the heart of inducing VILI during CV.

#### Gas exchange

When examined separately for each ventilation mode, only PaO_2_ of the CV group was PEEP dependent. Thus, even though raising the PEEP opened more regions and resulted in lower stiffness in the VV_N_ and CV_LB_ groups, it did not significantly improve PaO_2_. Nevertheless, the A-a gradient significantly improved by increasing the PEEP during VV_N_ implying that the higher PEEP also reduced ventilation/perfusion mismatch.

#### Surfactant protein and epithelial integrity

The levels of both SP-B and proSP-B highly depended on PEEP (Fig. 7). At PEEP6, SP-B in all ventilation modes dropped compared to unventilated mice suggesting that a static stretch higher than the physiological level corresponding to FRC hinders SP-B release. At PEEP3, SP-B during CV and CV_LB_ was lower while during VV_N,_ it was higher than the unventilated group in agreement with findings in normal guinea pigs [44]. Because proSP-B also increased at PEEP3 with VV_N_, this is evidence that variability in stretch amplitude delivered to the epithelium during VV_N_ triggers a dynamic mechanotransduction in alveolar epithelial type II cells that leads to the upregulation of both the release and production of SP-B even in severely injured lungs. Interestingly, while the proSP-B increased during CV, CV_LB_ was not able to increase proSP-B above the level of the unventilated group at PEEP3.

With regard to the integrity of the epithelium, we examined E-cadherin in the lavage fluid. The E-cadherin is a 120-kDa transmembrane glycoprotein localized to the lateral sides of epithelial cells linking them together and plays an important role in cell-cell mechanical signaling [45]. It is expressed in epithelial tissues but in the normal lung, it is not present in the alveolar liquid lining. Hence, appearance of the soluble fragments of E-cadherin in the lavage fluid is a clear indication of epithelial cell injury [42], [46], [47]. We found the 85 kDa soluble fragments of E-cadherin in the lavage fluid of unventilated animals which is likely due to the combined effects of HCl injury and the lavage process itself (Fig. 7C). Interestingly, however, while the higher PEEP was advantageous in terms of mechanics, it increased epithelial cell injury during CV and VV_N_. Unexpectedly, E-cadherin was not increased following CV at PEEP3. Perhaps the 60 min ventilation at PEEP3 was not sufficient in this mouse model of ALI to damage the epithelium beyond the unventilated group. On the other hand, stretch due to the intermittent large breaths in CV_LB_ without the compensating effects of variability started to break up the link between epithelial cells that showed up in the bulk biochemical assays even at PEEP3. Comparing the patterns of proSP-B and E-cadherin in Fig. 7B and C, we can see an apparent inverse relation at PEEP3: the highest level of proSP-B was associated with the lowest level of E-cadherin and vice versa. We can interpret this as follows. Increasing E-cadherin fragments in the lavage is likely due to overstretching the HCl-injured epithelium at least regionally. This in turn results in a breakup of E-cadherin anchoring epithelial cells. If the regional signal is strong enough, it will also show up in the bulk biochemical assays from the whole lung lavage. Furthermore, this breakup also leads to a loss of mechanical coupling among the cells. Since type II epithelial cells are stiffer than type I cells [48], weakening this mechanical interaction reduces the deformation of type II cells during lung inflation with subsequent reduction in surfactant secretion. We conclude that at least in this mouse model of ALI, increasing PEEP has a detrimental effect on surfactant and it does not protect the epithelium with likely consequences on longer term organ level physiology.

The implications of our results cannot be easily extrapolated to ventilating humans. We nevertheless note that a recent reanalysis of data from trials demonstrated that ventilating ARDS patients at a higher PEEP increased hospital survival whereas in patients without ARDS a lower PEEP tended to be more beneficial [49]. Regarding the gas exchange results (Fig. 5), the HCl treatment in this study is closer to a mild form of ARDS. However, ventilation in the human studies was carried out with CV and no epithelial markers were reported. While our results suggest that ventilation at PEEP6 leads to better organ level physiology, ventilation at PEEP3 leads to better epithelial protection. Whether or not these findings are specific to the mouse will need to be tested in future studies.

### Comparing the Performance of Ventilation Modes

#### Conventional ventilation vs alternative modes of ventilation

To better understand how alternative approaches improve lung physiology compared to CV, we computed the percent improvement at 60 min in some of the parameters during VV_N_ and CV_LB_ compared to CV. Briefly, at PEEP6, the improvements during VV_N_ and CV_LB_ were higher than at PEEP3. Moreover, at both PEEP levels, *H_min_* from the heterogeneous model and its percent increase were significantly improved during VV_N_ and CV_LB_ compared to CV. Therefore, VV_N_ and CV_LB_ could keep the most compliant regions of the lung open longer while those regions during CV gradually collapsed which resulted in higher peak airway pressures during CV. Both VV_N_ and CV_LB_ improved PaO_2_, %sO_2_ and A-a gradient at PEEP3 while at PEEP6, only VV_N_ was able significantly improve PaO_2_ and A-a gradient over CV. This might be due to a nonlinear synergistic effect between variability in VV_N_ and PEEP. Finally, the only parameter that was better during CV than CV_LB_ (but not VV_N_) was E-cadherin at PEEP3 (Fig. 7C). While the reason is unclear, we note that recruitment maneuvers were recently reported to increase inflammation and fibrogenic response with worsening lung function [50]. It is important to further investigate this phenomenon.

#### Comparing the performance of CV_LB_ and VV_N_

Funk et al. [23] has shown that the biologically variable ventilation improves oxygenation and lung compliance over conventional ventilation with recruitment maneuvers in a porcine model of lung injury. Nevertheless, in that study, the recruitment was delivered once every hour over a 5 hour ventilation period and it is unclear whether the RMs were matched to the largest breaths in VV. Since the effect of RM is transient in lung injury [21], it is likely that this frequency of RM is not enough to sustain an open lung. Indeed, Allen et al. [51] demonstrated that frequently delivered RM’s can improve gas exchange and lung mechanics over CV in normal mice. We constructed the large breaths in CV_LB_ so that they matched the 10 largest breaths of VV_N_ in 5 minutes both in size and timing with 1 large breath every 30 second, the same rate as in the study of Allen et al. [51]. Thus, our study constitutes a fair comparison of CV_LB_ and VV_N_.

**Figure 8:**
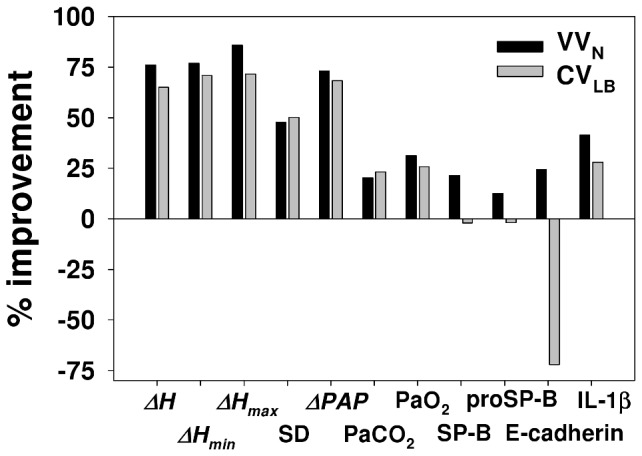
Comparison of ventilation modes. The graph compares the average improvements of VV_N_ and CV_LB_ over CV. The SD bar represents different experimental conditions including 2 PEEP levels and normal lung from our previous study [27]. Only those parameters are included for which either CV_LB_, VV_N_ or both significantly improved over CV. See text for more explanation.

We also examined the mean airway pressure at PEEP3 and found that at 60 min, there was no difference between CV_LB_ and VV_N_ (data not shown). However, at 60 min, *H* (Fig. 2) and *ΔPAP* (Fig. 4) at PEEP6 were lower during VV_N_ than CV_LB_. Additionally, PaO_2_ and A-a gradient at PEEP6 was better during VV_N_ but not CV_LB_ than CV. The likely reason is that CV_LB_ could not improve over CV due to inter-animal variability which was overcome by VV_N_ suggesting that VV_N_ is more robust. In order to further explore the differences between these two modes of ventilation, we compiled data in all parameters for which there was a statistically significant improvement of CV_LB_ and/or VV_N_ over CV. The results are summarized in Fig. 8. Since the VV_N_ and CV_LB_ were identical in our previous study [27], we also included physiology data from normal mice as well as IL-1β as an indicator of lung injury. In Fig. 8, each bar represents a single number, the percent improvement in that parameter. For example, the first bar was calculated by taking the relative difference of the means of *ΔH* at 60 min during VV_N_ and CV for a given condition such as the data at PEEP3. Next, this improvement was calculated and averaged for all available conditions including PEEP3, PEEP6 as well as PEEP3 in normal mice from our previous study [27]. The second bar was obtained similarly but now for CV_LB_. Thus, the corresponding bars for each parameter represent paired data and a comprehensive comparison of VV_N_ and CV_LB_ can be obtained by using a non-parametric paired t-test. The median improvements of CV_LB_ and VV_N_ over CV were 28% and 41%, respectively (p = 0.005). It is also noteworthy that VV_N_ but not CV_LB_ caused significantly less inflammatory response than CV as indicated by significantly lower level of IL-1β and CV_LB_ also caused more epithelial injury than CV (p<0.01).

**Figure 9:**
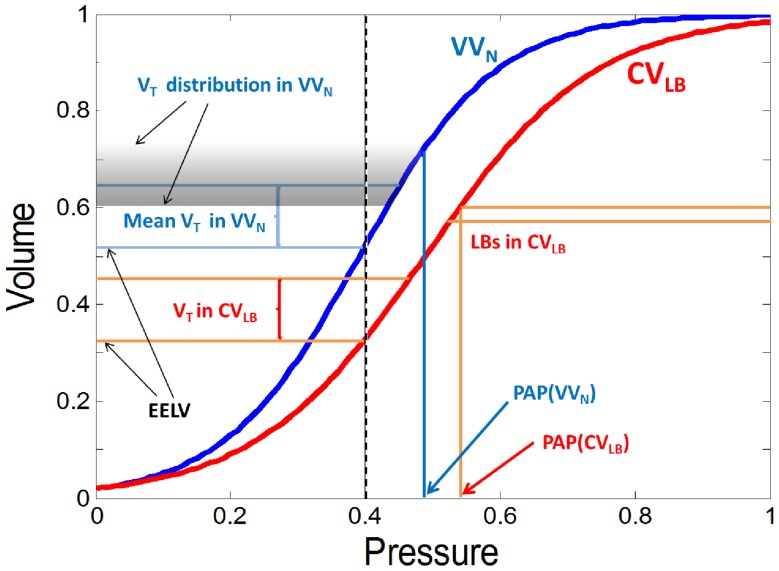
Schematic representation of ventilation along the normalized pressure-volume curve during CV_LB_ and VV_N_. The vertical dashed black line at 0.4 represents PEEP. The intersections of PEEP and the pressure-volume curves mark the end-expiratory lung volumes (EELV) during the two ventilation modes upon which V_T_ is superimposed. For CV_LB_ (red), we also show the large breaths (LB) and the corresponding peak airway pressure (PAP). For VV_N_ (blue), there is a range of V_T_s superimposed on EELV. The corresponding end-inspiratory volumes have a distribution shown by the shaded area. The probability of a given tidal volume is proportional to the gray scale. Also notice that the mean V_T_ in VV_N_ is the same as in CV_LB_, but the distribution of V_T_s goes below the V_T_ of CV_LB_ and stretches up to the LBs in CV_LB_.

Since both VV_N_ and CV_LB_ had already reached their plateau in every mechanical parameter by 30 minutes of ventilation, we combined all the data points for each parameter from 30 minutes to 60 minutes in the CV_LB_ and VV_N_ groups. At both PEEPs, the plateau levels of *H*, *ΔH* and *ΔPAP* were significantly higher during CV_LB_ than VV_n_. This suggests that VV_N_ better recruits the lung independent of PEEP which we attribute to the presence of intermediate V_T_s in agreement with previous computer model-based predictions [52]. The pressure-volume curve of the lung during VV_N_ must therefore be different than during CV_LB_ and ventilation is superimposed on different lung volumes with different compliance. This scenario is summarized schematically in Fig. 9. We can see that the V_T_s are superimposed on the same PEEP (dashed line) but different end-expiratory lung volumes (EELV) which results in a higher compliance (slope of the pressure-volume curve) and a lower peak airway pressure (PAP) during VV_N_. At the start of ventilation (time 0), the pressure-volume curve would be somewhat to the left of the VV_N_ curve (blue) whereas after 60 min of ventilation with CV, the pressure-volume curve would be to the far right of the CV_LB_ curve (red). Thus, better and safer stretching of the lung and especially the epithelium with reduced repetitive opening and closing during VV_N_ eventually results in molecular changes including increased surfactant and less injury such as lower level of soluble E-cadherin.

To summarize, our first conclusion is that to the extent that our results are not specific to mice, there is room for improvement on current clinical ventilation approaches. Second, PEEP had a significant effect on the performance of all ventilation methods. With regard to physiology, the higher PEEP protected the lung from collapse and reduced tissue heterogeneity. However, the lower PEEP better protected the epithelium and had a positive effect on surfactant especially during VV. Thus, selection of optimal PEEP should be based on balancing organ level physiology with epithelial injury. Third, VV better maintains an open lung, which allows ventilation at a lower PEEP with lower maximum lung stretch reducing epithelial injury. Additionally, the dynamic mechanotransduction induced by VV has a beneficial effect on surfactant metabolism.
